# Comparative evaluation of semi-quantitative CT-severity scoring versus serum lactate dehydrogenase as prognostic biomarkers for disease severity and clinical outcome of COVID-19 patients

**DOI:** 10.1186/s43055-021-00493-2

**Published:** 2021-04-28

**Authors:** Ahmed M. Magdy, Mohammad A. Saad, Ahmed F. El Khateeb, Mona I. Ahmed, Dina H. Gamal El-Din

**Affiliations:** 1 Radiology department, Faculty of Medicine, Fayoum University, Fayoum, Egypt; 2 Department of critical care, Faculty of Medicine, Fayoum University, Fayoum, Egypt; 3 Department of chest disease and tuberculosis, Faculty of Medicine, Fayoum University, Fayoum, Egypt; 4 Radiology Department, Faculty of Medicine, Cairo University, Cairo, Egypt

**Keywords:** Coronavirus disease 2019 (COVID-19), Prognostic biomarker, CT severity scoring, LDH

## Abstract

**Background:**

Coronavirus disease 2019 pandemic causes significant strain on healthcare infrastructure and medical resources. So, it becomes crucial to identify reliable predictor biomarkers for COVID-19 disease severity and short term mortality. Many biomarkers are currently investigated for their prognostic role in COVID-19 patients. Our study is retrospective and aims to evaluate role of semi-quantitative CT-severity scoring versus LDH as prognostic biomarkers for COVID-19 disease severity and short-term clinical outcome.

**Results:**

Two hundred sixty-six patients between April 2020 and November 2020 with positive RT-PCR results underwent non-enhanced CT scan chest in our hospital and were retrospectively evaluated for CT severity scoring and serum LDH level measurement. Data were correlated with clinical disease severity. CT severity score and LDH were significantly higher in severe and critical cases compared to mild cases (*P* value < 0.001). High predictive significance of CT severity score for COVID-19 disease course noted, with cut-off value ≥ 13 highly predictive of severe disease (96.96% accuracy); cut-off value ≥ 16 highly predictive of critical disease (94.21% accuracy); and cut-off value ≥ 19 highly predictive of short-term mortality (92.56% accuracy). CT severity score has higher sensitivity, specificity, positive, and negative predictive values as well as overall accuracy compared to LDH level in predicting severe, critical cases, and short-term mortality.

**Conclusion:**

Semi-quantitative CT severity scoring has high predictive significance for COVID-19 disease severity and short-term mortality with higher sensitivity, specificity, and overall accuracy compared to LDH. Our study strongly supports the use of CT severity scoring as a powerful prognostic biomarker for COVID-19 disease severity and short-term clinical outcome to allow triage of need for hospital admission, earlier medical interference, and to effectively prioritize medical resources for cases with high mortality risk for better decision making and clinical outcome.

## Background

COVID-19 (SARS-CoV-2) was initially described in Wuhan, China and was declared to be a global health emergency on 30 January 2020 [1].

Clinical disease severity score is classified into mild, severe, and critical according to Chinese center of disease control (CDC) [2].

Thin-slice chest CT plays a crucial role in COVID-19 early detection and assessment of disease burden [2]. CT was reported to have high sensitivity in patients infected by SARS-CoV-2, so it is largely used to help patient management [3].

Chest CT is a non-invasive, conventional imaging modality with high speed and accuracy [4].

Chest computed tomography severity score (CT-SSS) by Yang et al. and Pan et al. [5, 6] was published in “Radiology” in 2020. It was created to help assess COVID-19 pulmonary affection burden and uses lung opacification as an equivalent for extension of the disease in the lungs.

LDH is an intracellular enzyme in almost all organ systems, which catalyzes the conversion of pyruvate and lactate [7]. The enzyme is formed of two subunits (A and B). In humans, it is present in five separate isozymes (LDH-1 in cardiomyocytes, LDH-2 in reticuloendothelial system, LDH-3 in pneumocytes, LDH-4 in the kidneys and pancreas, and LDH-5 in the liver and striated muscles). Severe infections can cause cytokine-mediated tissue damage and then LDH release and elevated levels. As LDH is present within pneomocytes (isozyme 3), severe COVID-19 cases may be expected to release greater amounts of LDH [8].

Lactate dehydrogenase (LDH) is one of the biomarkers under investigation for its role in prediction of COVID-19 patients’ prognosis. Compared to other prognostic laboratory biomarkers for COVID-19 disease severity including CRP, lymphocytes, and AST, LDH was found to have higher accuracy and greater area under the curve (AUC) for predicting COVID-19 disease severity [9–11].

Also, COVID-19 can cause injury to different organs such as the liver, kidney, and heart which when affected can cause further elevation in LDH levels [12–16].

The aim of our study is comparative evaluation of semi-quantitative CT-severity scoring versus serum LDH as prognostic biomarkers for disease severity and clinical outcome of COVID-19 patients

## Methods

### Patients

This study is a single-center retrospective analysis; a total of 266 patients referred from the chest clinic and emergency department in our university hospital were enrolled between April 2020 and November 2020, with clinical suspicion of COVID-19 pneumonia. They showed positive RT-PCR results. The patients underwent non-enhanced MSCT scan of the chest and serum LDH level measurement in the same day done between 5 and 10 days from the onset of symptoms. The local ethical committee approved this retrospective study.

#### Inclusion criteria

PCR-positive COVID-19 cases isolated in our hospital.

#### Exclusion criteria

Patients less than 18 years old.

Pregnant females.

Patients with significant artifacts on MSCT images.

### Methods

All patients were subjected to:

- ❖ Full history taking.
- ❖ LDH level assessment with an upper limit cut-off 255 U/L.
- ❖ Clinical disease severity scoring was evaluated for all cases, based on the criteria provided by the Chinese Center of Disease Control (CDC) as follows [2]: mild disease including non-pneumonia or mild pneumonia (mild symptoms without dyspnea; respiratory rate < 30/min and blood oxygen saturation (SpO2) > 93); severe disease presenting with dyspnea, respiratory rate ≥ 30/min, and SpO2 ≤ 93%; critical disease including adult respiratory distress syndrome (ARDS) or respiratory failure, multiple organ dysfunction (MOD) or failure (MOF), or septic shock.
- ❖ Non-enhanced CT of the chest:
  - Single MSCT scanner (Toshiba Aquilion Prime 160; Toshiba medical systems, Japan) used for examining all patients.
  - All patients were scanned in the supine position during inspiratory breath hold with the range of the scans were from the root of the neck to the level of the upper pole of the right kidney (cranio-caudally). The detailed parameters for MSCT acquisition were 120 kVp, 100–300 mAs, pitch 1–1.5, collimation 0.625–5 mm, and slice thickness 1–3 mm. Sharp reconstruction algorithm was applied. No IV contrast was administrated.
  - Appropriate infection control parameters were arranged consisting of appropriate sanitation of MDCT facilities and patient’s isolation.
  - The studies were sent to, processed, and reviewed on PACS system (Paxera Ultima version 6.0.0.1).
  - The volumetric MSCT chest images were reviewed on both lung window (1500 WW and − 500 WL) and mediastinal window (400 WW and 60 WL) settings.
  - The CT chest images were processes and reconstructed in axial, coronal, and sagittal planes (multi-planar reconstruction; MPR). Semi-quantitative color-coded images for all cases were reviewed using Sante DICOM Viewer Pro to be processed and manipulated.
  - Two chest radiologists (with 13 years of experience in interpreting chest CT images) independently evaluated all patients, blinded to clinical characteristics and laboratory data. In case of discrepancy, studies were re-reviewed by the third chest radiologist with 25 years of experience then findings were discussed to reach a general agreement.
  - The chest CT scans were evaluated for the following:
    - Presence of pulmonary parenchymal lesions, ground glass opacity (GGO), pulmonary consolidation, septal thickening (crazy-paving pattern), halo and reversed halo sign, pulmonary nodules and masses, cavitations, and tree-in-bud-pattern.
    - Pulmonary parenchymal lesions distribution (laterality, distribution predilection within the lobes (central, peripheral/subpleural, central, and peripheral).
    - Presence of associated extra-pulmonary chest lesions (including pleural thickening, pleural effusion or significant pathologically enlarged hilar or mediastinal lymphadenopathy).
    - Identification of CT-severity score for evaluation of pulmonary affection burden in all cases using a semi-quantitative CT severity scoring system (CT-SSS) proposed by Yang et al. and Pan et al. [5, 6]. This scoring system was calculated per each lobe of the 5 lobes of both lungs regarding the extent of pathologic involvement: score 0, no parenchymal involvement; score 1, < 5% parenchymal involvement; score 2, 5–25% parenchymal involvement; score 3, 26–50% parenchymal involvement; score 4, 51–75% parenchymal involvement; and score 5, > 75% parenchymal involvement. The resulting global CT score was the sum of each individual lobar score from 0 to 25.

### Statistical analysis

Data were coded and entered using the statistical package SPSS (Statistical Package for the Social Sciences) version 26 (IBM Corp., Armonk, NY, USA). Data was summarized using mean, standard deviation, median, minimum and maximum in quantitative data, and using frequency (count) and relative frequency (percentage) for categorical data. Standard diagnostic indices including sensitivity, specificity, positive predictive value (PPV), negative predictive value (NPV), and diagnostic efficacy were calculated. ROC curve was constructed with area under curve analysis performed to detect best cut-off value of CT-SSS and LDH for detection of severe cases. Comparisons between quantitative variables were done using the non-parametric Kruskal-Wallis and Mann-Whitney tests. *P* value less than 0.05 was considered as statistically significant.

## Results

The study population included 266 patients (176 males, 90 females; mean age was 34.75 ± 10.7 years).

Regarding presence of medical comorbidities, the percent of cases with at least one comorbidity (chronic chest or heart disease, diabetes, hypertension) was 33.7%.

**Fig. 1:**
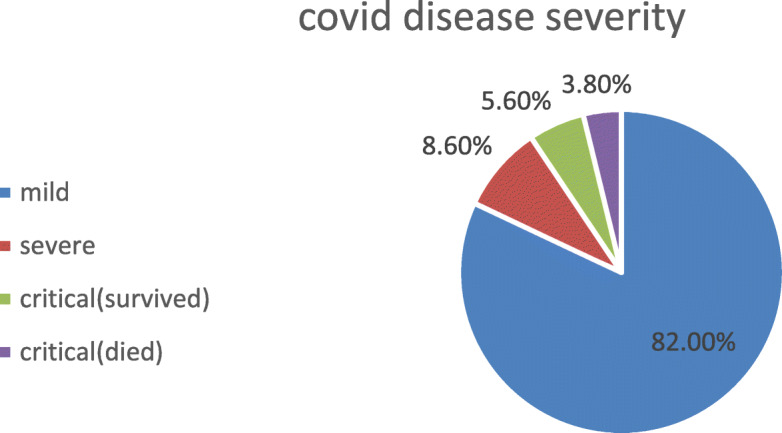
Disease severity distribution

The clinical disease severity distribution of our patients: mild cases 218 (82.0%), severe cases 23 (8.6%), and critical cases 25 (9.4%). From the critical cases, 10 cases died (3.8%) (Fig. 1).

The average age of severe and critical cases was 41.23 ± 14.38 years which was significantly higher than that of non-severe cases 32.11 ± 10.51 years (*P* value 0.009). Patients with at least one medical comorbidity were more likely to have severe and critical disease course (*P* value < 0.05).

**Fig. 2:**
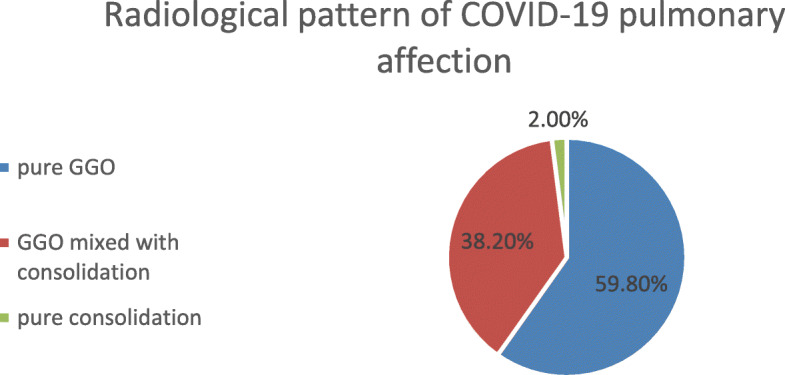
Radiological pattern of COVID-19 pulmonary affection

MSCT scan images were evaluated and analyzed for all examined cases. Twenty patients showed normal CT chest with no radiological signs of pulmonary affection. In the remaining 246 patients, ground glass opacities were the cardinal radiological feature, with pure ground glass opacities detected in 147 patients (59.8% of cases with positive pulmonary affection), ground glass opacities mixed with areas of consolidation in 94 patients (38.2%), and pure consolidation detected in only 5 patients (2.0%) (Fig. 2).

**Fig. 3:**
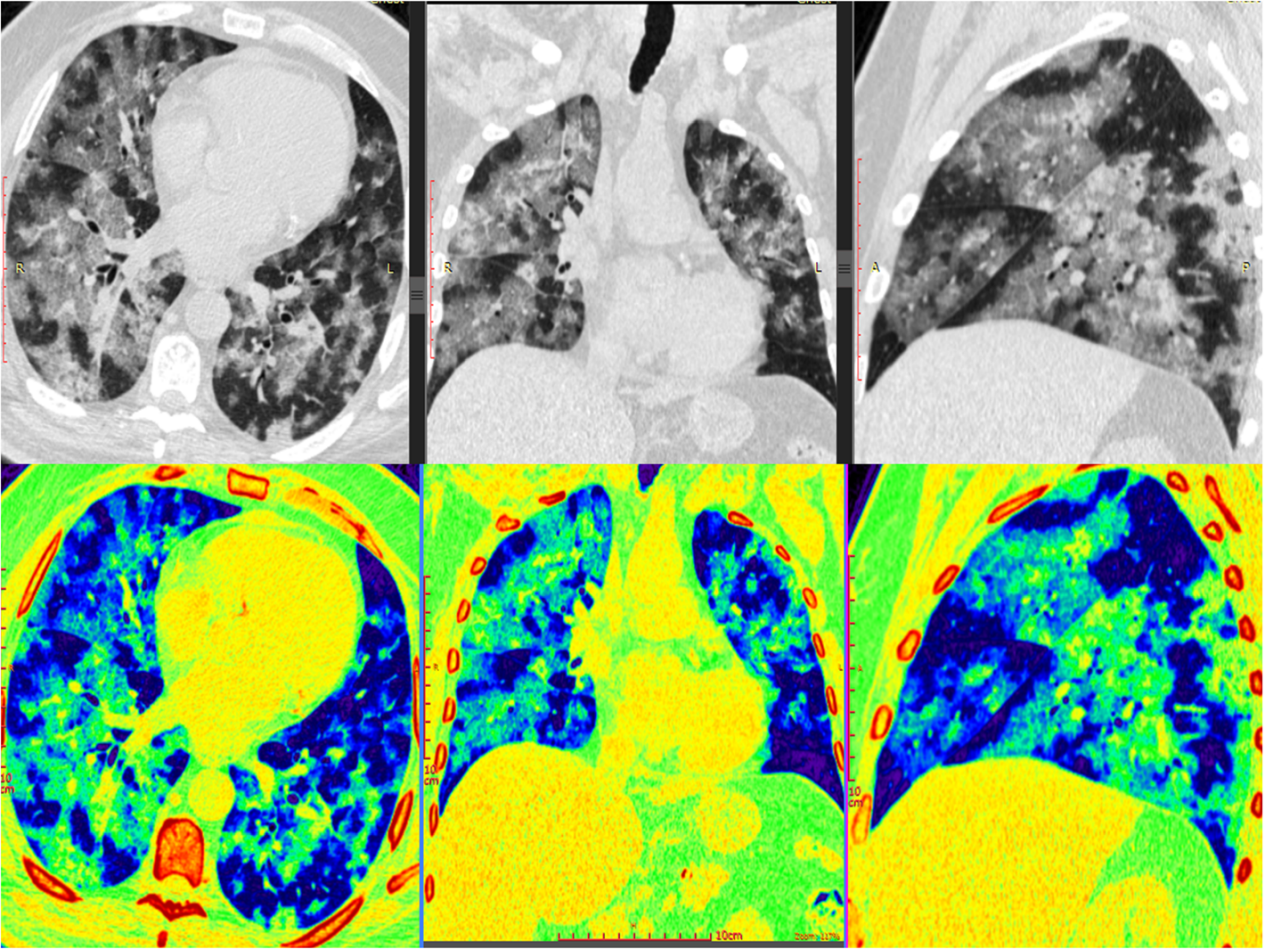
A 55-year-old female patient presented with fever, dyspnea, and cough. MSCT scan with multi-planar and color-coded images showed widespread confluent bilateral peripheral and central predominant ground glass opacities with septal and vascular thickening. CT-SSS was 19 and serum LDH was 550. The patient exhibited critical disease course with ICU admission, invasive mechanical ventilation, and finally died

**Fig. 4:**
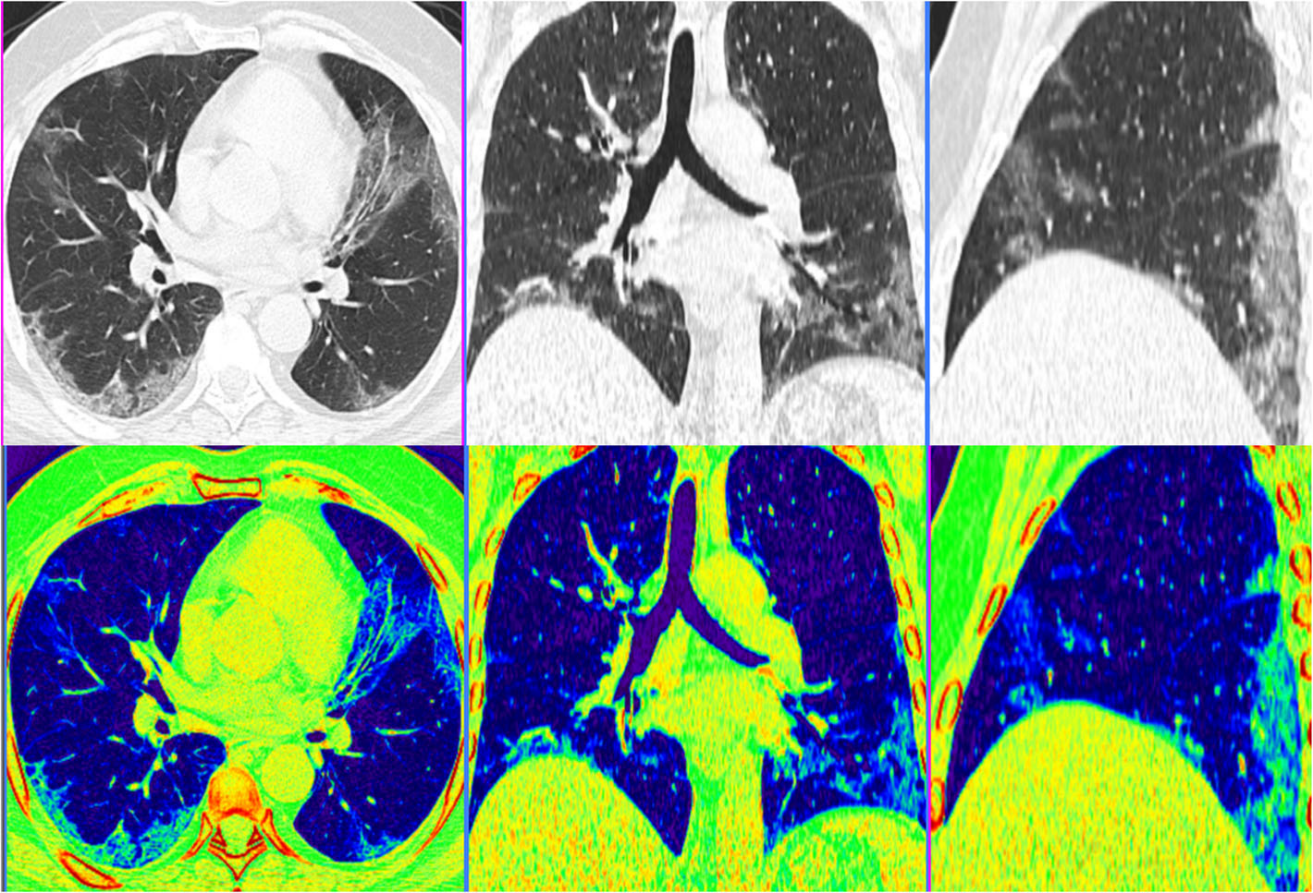
A 48-year-old male patient presented with fever and cough. MSCT scan with multi-planar and color-coded images showed multi-focal bilateral predominantly peripheral subpleural patchy ground glass opacities with septal and vascular thickening. CT-SSS was 11 and serum LDH was 239. The patient exhibited non-severe disease course

**Fig. 5:**
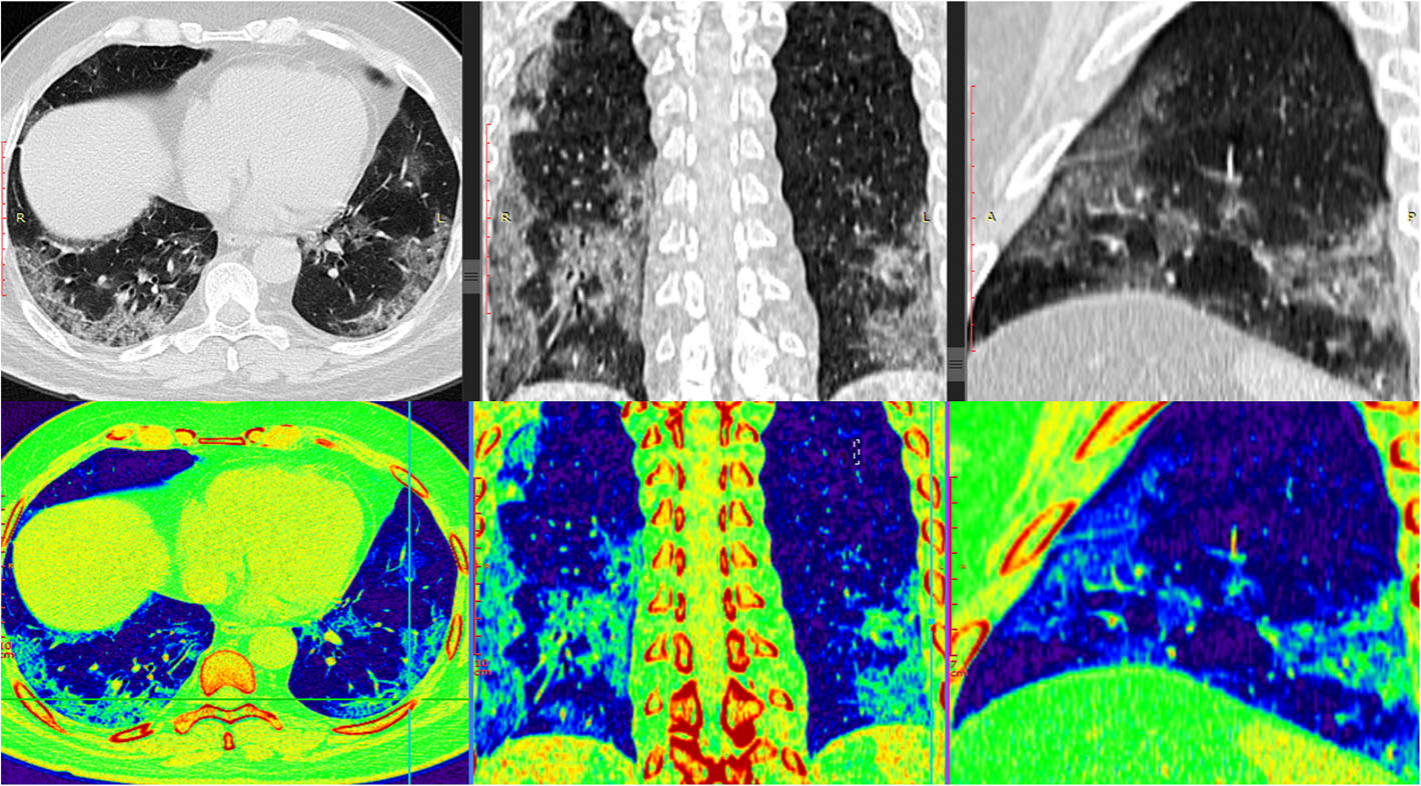
A 51-year-old male patient presented with fever, cough, and dyspnea. MSCT scan with multi-planar and color-coded images revealed widespread bilateral predominantly peripheral subpleural patchy and confluent ground glass opacities with septal and vascular thickening and reverse halo “atoll sign“. CT-SSS was 15 and serum LDH was 632. The patient exhibited severe disease course with ICU admission for 12 days followed by clinical improvement and discharge

Most of the cases with positive pulmonary affection display bilateral pulmonary involvement (79%) with peripheral/subpleural predilection (89%) (Figs. 3, 4, and 5). Mild to moderate pleural effusion noted in 5 patients (2.03%). No tree-in-bud pattern, pulmonary cavitation, mass-like lesions, or significant lymphadenopathy could be detected.

Patients with both central and peripheral pulmonary distribution, crazy-paving pattern, consolidation, and pleural effusion were more likely to have severe and critical disease course (*P* value <0.001).

The CT severity score in our study ranged from 0 to 22, with a mean value of 6.89 ± 6.18. Serum LDH levels ranged from 19.6 to 2121 with a mean value of 428.68 ± 292.76 (Table 1).

**Table 1:** CT-SSS and LDH spectrum in the study

	Mean	Standard deviation	Median	Minimum	Maximum
**CT-Severity scoring system (CT-SSS)**	6.89	6.18	4.00	0.00	22.00
**LDH**	428.68	292.76	328.00	19.6	2121.00

The CT severity score and LDH levels were significantly higher in severe and critical cases as well as in those who died compared to mild cases with *P* value < 0.001 (Table 2). No statistical significance detected in CT severity score and LDH levels between severe and critically severe cases (*P* value 0.963 and 0.569, respectively).

**Table 2:** Correlation between CT-SSS, LDH, and disease severity

		COVID disease severity				
		Mild	Severe	Critical	Died	***P*** value
**CT-severity scoring system (CT-SSS)**	**Mean**	3.57	12.48	18.00	20.10	< 0.001
	**Standard deviation**	3.70	4.19	2.36	1.10	
	**Median**	2.00	13.00	18.00	20.00	
	**Minimum**	0.00	0.00	14.00	18.00	
	**Maximum**	12.00	21.00	21.00	22.00	
**LDH**	**Mean**	311.08	467.06	706.73	875.40	< 0.001
	**Standard deviation**	146.48	231.06	293.68	502.99	
	**Median**	278.00	429.00	800.00	794.00	
	**Minimum**	2.60	19.00	221.00	370.00	
	**Maximum**	1031.00	853.00	1263.00	2121.00	

**Fig. 6:**
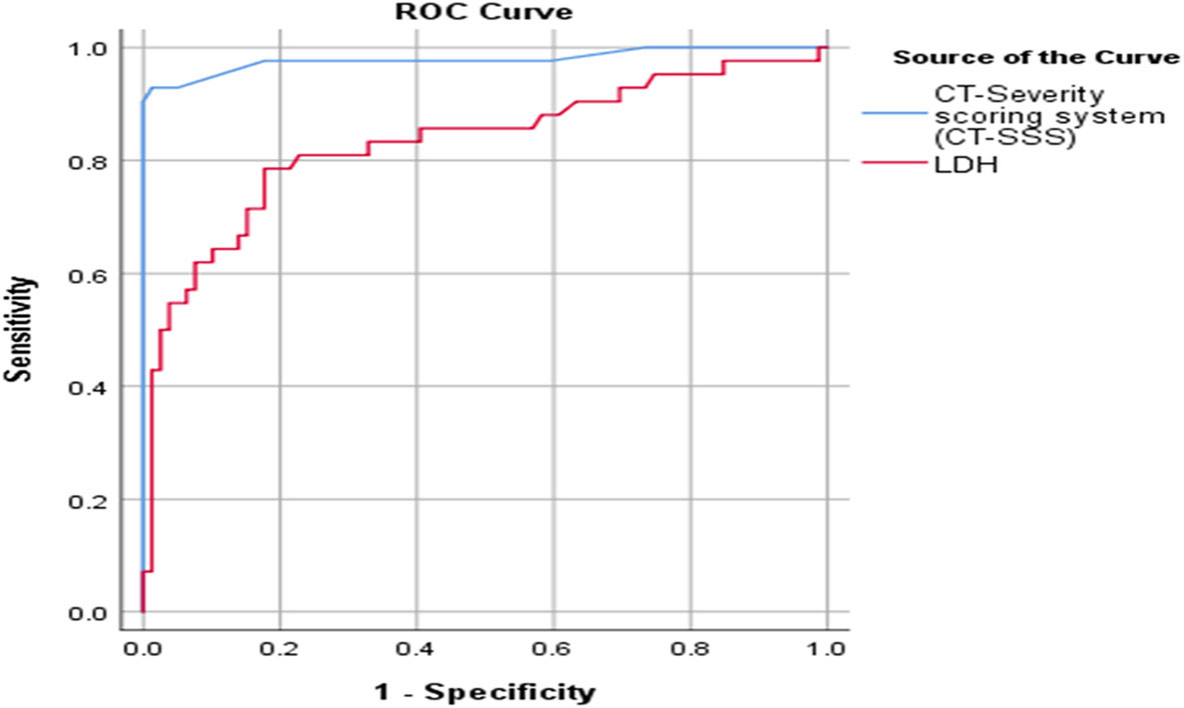
ROC curve for severe cases using CT-SSS and LDH

The ROC curve analysis showed that the area under curve (AUC) was significantly high using CT-severity score cut-off point ≥ 13 and serum LDH level cut-off point ≥ 386 U/L for severe COVID-19 cases, with sensitivity, specificity, positive predictive value, negative predictive value, and accuracy of 92.9%, 98.7%, 97.5%, 96.3%, and 96.96% compared to 78.6%, 82.3%, 70.21%, 87.84, and 80.99%, respectively (Fig. 6, Table 3).

**Table 3:** ROC curve cut-off value for severe cases using CT-SSS and LDH

	Area under curve	***P*** value	95% confidence interval		Cut-off value	Sensitivity (%)	Specificity (%)	PPV (%)	NPV (%)	Accuracy (%)
			Lower bound	Upper bound						
**CT-severity scoring system (CT-SSS)**	0.979	< 0.001	0.947	1.010	12.5	92.9	98.7	97.50	96.30	96.69
**LDH**	0.832	< 0.001	0.747	0.917	386	78.6	82.3	70.21	87.84	80.99

**Fig. 7:**
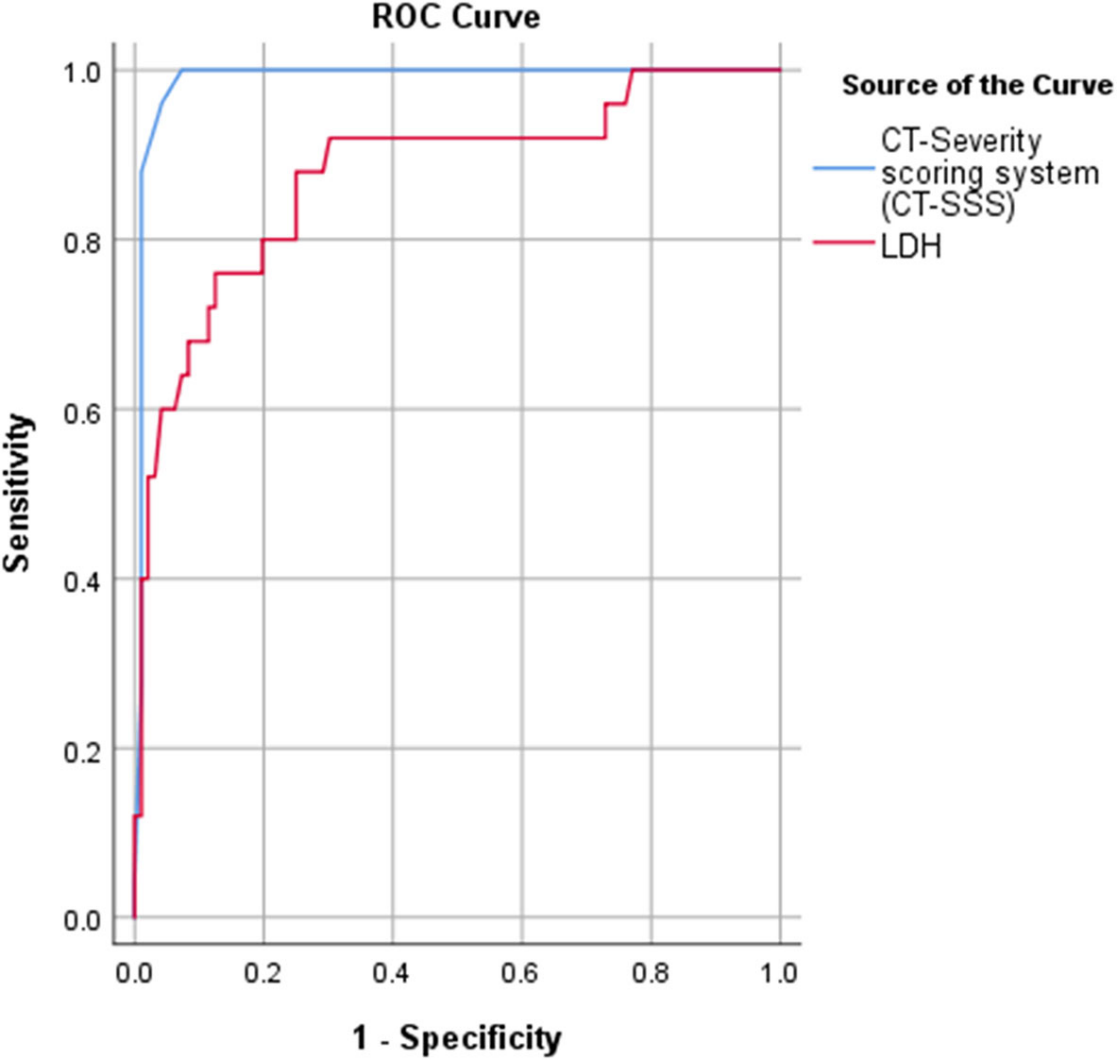
ROC curve for critical cases using CT-SSS and LDH

Also, the ROC curve analysis showed that the area under curve (AUC) was significantly high using CT-severity score cut-off point ≥ 16 and serum LDH level cut-off point ≥ 400 U/L for critical COVID-19 cases, with sensitivity, specificity, positive predictive value, negative predictive value, and accuracy of 100%, 92.7%, 78.13%, 100%, and 94.21% compared to 76%, 87.5%, 61.29%, 93.33, and 85.12% respectively (Fig. 7, Table 4).

**Table 4:** ROC curve cut-off value for critical cases using CT-SSS and LDH

	Area under the ROC curve	***P*** value	95% confidence interval							
			Lower bound	Upper bound	Cut-off	Sensitivity %	Specificity %	PPV (%)	NPV (%)	Accuracy (%)
**CT-severity scoring system (CT-SSS)**	0.988	< 0.001	0.970	1.006	15.5	100	92.7	78.13	100.00	94.21
**LDH**	0.876	< 0.001	0.791	0.962	399.5	76	87.5	61.29	93.33	85.12

**Fig. 8:**
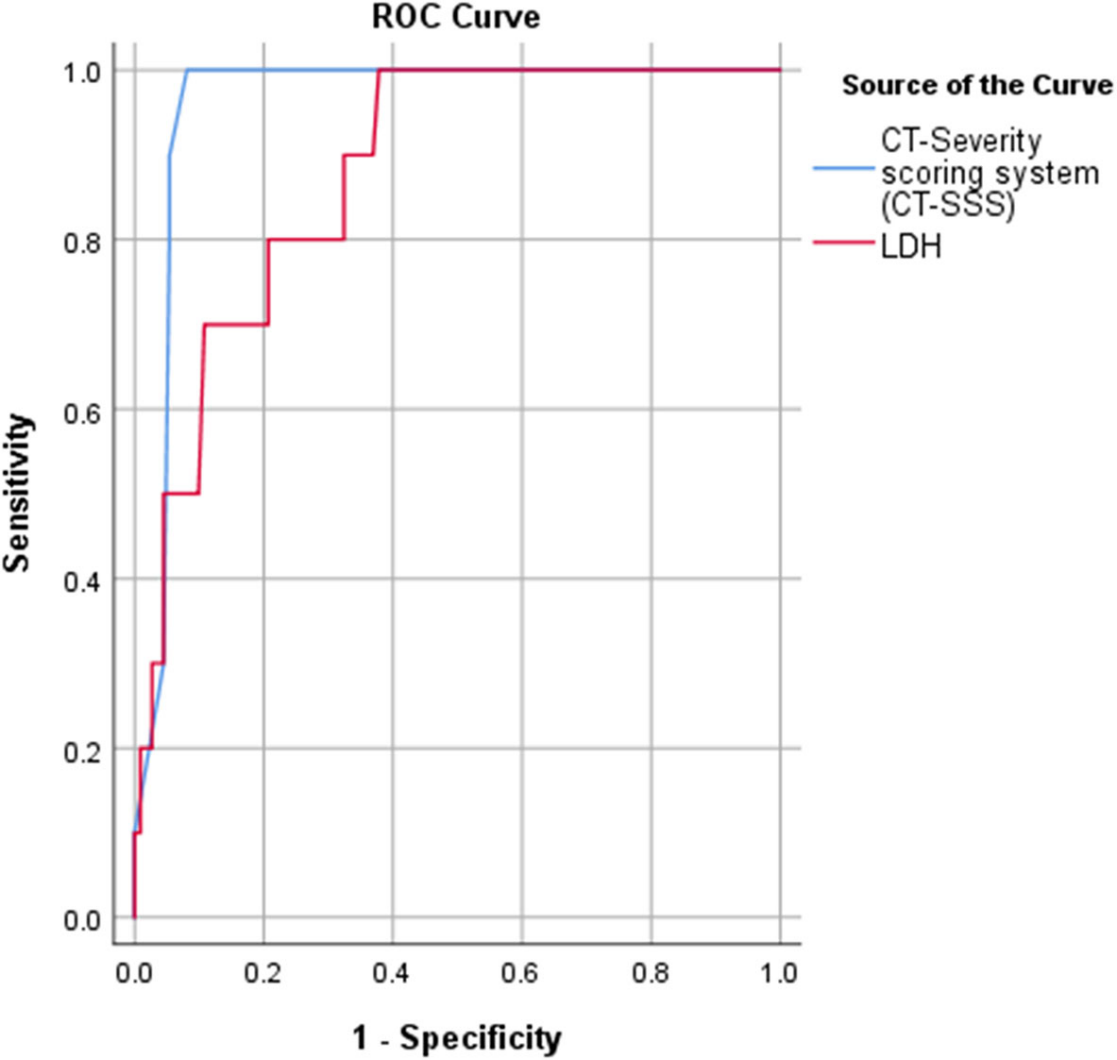
ROC curve for COVID-19 mortality using CT-SSS and LDH

Additionally, the ROC curve analysis showed that the area under curve (AUC) was significantly high using CT-severity score cut-off point ≥ 19 and serum LDH level with cutoff point ≥ 534 U/L for COVID-19 cases mortality, with sensitivity, specificity, positive predictive value, negative predictive value, and accuracy of 100%, 91.9%, 52.63%, 100%, and 92.56% compared to 100%, 62.2%, 19.23%, 100%, and 65.29%, respectively (Fig. 8, Table 5).

**Table 5:** ROC curve cut-off value for COVID-19 mortality using CT-SSS and LDH

	Area under the curve	***P*** value	95% confidence interval							
			Lower bound	Upper bound	Cut-off	Sensitivity %	Specificity %	PPV (%)	NPV (%)	Accuracy (%)
**CT-severity scoring system (CT-SSS)**	0.959	< 0.001	0.924	0.993	18.5	100	91.9	52.63	100.00	92.56
**LDH**	0.876	< 0.001	0.788	0.964	533.5	100	62.2	19.23	100.00	65.29

## Discussion

Coronavirus disease 2019 is highly infectious with higher fatality rate than other respiratory tract viral infectious diseases. Many biomarkers are currently investigated for their prognostic role in COVID-19 patients’ disease severity and mortality [9–11].

CT chest parenchymal assessment may reflect short-term outcome in COVID-19 cases, providing a direct visualization of anatomic injury [17].

CT scoring system proposed by Yang et al. and Pan et al. [5, 6] for COVID-19 patients could help in assessment of pulmonary disease burden and had predictive value for clinical disease severity [18].

Lactate dehydrogenase (LDH) is an intracellular enzyme found in almost all organ systems. Elevated serum LDH level is present in many diseases including COVID-19. Compared to other laboratory biomarkers including CRP, lymphocytes, and AST for their predictive value in COVID-19 disease severity and clinical outcome, LDH was found to have higher accuracy and greater area under the curve (AUC) [9–11].

The aim of our study is comparative evaluation of semi-quantitative CT-severity scoring versus serum LDH as prognostic biomarkers for disease severity and clinical outcome of COVID-19 patients.

A total of 266 patients were enrolled in our study with positive RT-PCR results. The patients underwent non-contrast CT scan of the chest and serum LDH level measurement in the same day, done between 5 and 10 days from the onset of symptoms.

The clinical disease severity distribution of our patients: mild cases 218 (82.0%), severe cases 23 (8.6%), and critical cases 25 (9.4%). From the critical cases, 10 cases died (3.8%).

The average age of severe and critical cases was significantly higher than that of non-severe cases (*P* value 0.009). Patients with at least one medical comorbidity (chronic chest or heart disease, diabetes, hypertension) were more likely to have severe and critical disease course (*P* value < 0.05). Therefore, more attention should be paid to elderly COVID-19 patients with comorbidities at admission in clinical practice to improve the outcome. This agreed with the study of Ioannidis et al. [19] which found that elderly individuals are more likely to have severe COVID-19 disease course and increased mortality risk.

The CT features of COVID-19 pneumonia in our study are compatible with that published in the literature [20–23]. Ground glass opacities were the most common CT chest radiological finding with bilateral peripheral/subpleural predilection. Tree-in-bud pattern, pulmonary cavitation, mass-like lesions, significant lymphadenopathy, and pleural effusion were rare to be encountered. Also, this study agreed with Wang et al. [24] who noticed ground glass opacities to be the commonest CT feature in COVID-19 patients; it persisted till the late absorption stage and became the last radiological finding to resolve.

We found that patients with both central and peripheral pulmonary distribution, crazy-paving pattern, consolidation, and pleural effusion were more likely to have severe and critical disease course (*P* value < 0.001) which is compatible with the study of Yuan et al. [25] which stated that these radiological findings were significantly associated with adverse clinical outcome.

In our study, we found that CT severity score and LDH levels in severe cases and critical cases were significantly higher compared to mild cases with *P* value < 0.001. No statistical significance detected in CT severity score and LDH levels between severe and critically severe cases.

This agreed with the study done by Francone, et al. [17] which stated that CT severity scores were significantly lower in the mild disease category compared to severe/critical disease categories confirming high correlation between the radiological findings and clinical stages. This is also compatible with Liu et al. [26] who noticed higher CT severity score in COVID-19 patients with severe/critical disease course compared to patients with moderate disease.

This study agreed with the pooled analysis of Henry et al. [16] which stated that there is an association between elevated LDH values and worse outcomes in patients with COVID-19; increased LDH levels were associated with about ~ 6-fold increase in odds of developing severe/critical disease. LDH level was found to be an important tool in determining prognosis. Lv et al [27] also demonstrated that high LDH levels were positive predictors of an adverse outcome in severe/critical COVID-19 cases.

By ROC curve analysis in our study, we were able to confirm high predictive significance of CT severity score for COVID-19 short-term clinical outcome, with cut-off value ≥ 13 highly predictive of severe disease with sensitivity, specificity, and accuracy of 92.9%, 98.7%, and 96.96%, respectively; cut-off value ≥ 16 highly predictive of critical disease with sensitivity, specificity, and accuracy of 100%, 92.7%, and 94.21%, respectively; and cut-off value ≥ 19 highly predictive of short-term mortality with sensitivity, specificity, and accuracy of 100%, 91.9%, and 92.56%, respectively. This study showed that CT severity scoring had significantly higher prognostic value compared to serum LDH for COVID-19 disease severity and short-term clinical outcome with higher sensitivity, specificity, and overall accuracy.

This agreed with Francone et al. [17] who found that CT parenchymal affection may more accurately correlate with short-term disease outcome compared with other inflammatory biomarkers. He also stated that CT-severity score of ≥ 18/25 is highly predictive of mortality in COVID-19 patients’ short-term follow-up. In addition; according to Zhou et al. [28] the optimal CT-severity score cut-off value of 16.5/25 points had 69.4% sensitivity and 82.3% specificity for predicting poor prognosis in COVID-19 patients.

## Conclusion

Semi-quantitative CT severity scoring has high predictive significance for COVID-19 disease severity and short-term mortality with higher sensitivity, specificity, and overall accuracy compared to LDH. Our study strongly supports the use of CT severity scoring as a powerful prognostic biomarker for COVID-19 disease severity and short-term clinical outcome to allow triage of need for hospital admission, earlier medical interference and to effectively prioritize medical resources for cases with high mortality risk for better decision making and clinical outcome.

Our study had some limitations. First of all, our single-center study only included non-pregnant adults. The generalizability of results of this study in pregnant women and children infected with COVID-19 is not clear. Larger future multi-center studies are needed to confirm the generalizability of this study on a larger scale and its impact on clinical performance and decision-making in COVID-19 pandemic. Second, although the semi-quantitative visual assessment of CT severity score used in our study was proved to be a relatively reliable method for assessment of lung disease burden, future studies with application of artificial intelligence-assisted technology could help to increase reproductivity and accuracy of quantitative evaluation. Finally, our study evaluated the prognostic value of only one promising laboratory biomarker which is serum LDH level. Future studies are recommended to investigate the prognostic significance of other clinical and laboratory biomarkers for COVID-19 clinical outcome.

## Data Availability

The datasets used and/or analyzed during the current study are available from the corresponding author on reasonable request.

## Abbreviations

2019-COV: 2019 coronavirus
ARDS: Acute respiratory distress syndrome
CDC: Chinese center of disease control
COVID-19: Corona virus disease 19
CT: Computed tomography
CT-SSS: Computed tomography severity scoring system
GGO: Ground glass opacity
LDH: Lactate dehydrogenase
MDCT: Multi-detector computed tomography
MOF: Multiple organ failure
MPR: Multi-planar reconstruction
MSCT: Multi-slice computed tomography
NPV: Negative predictive value
PCR: Polymerase chain reaction
PPV: Positive predictive value
RT-PCR: Reverse transcriptase polymerase chain reaction
SARS-Cov-2: Severe acute respiratory syndrome coronavirus 2
SpO2: Blood oxygen saturation
