# Correction to: Comparative evaluation of semi-quantitative CT-severity scoring versus serum lactate dehydrogenase as prognostic biomarkers for disease severity and clinical outcome of COVID-19 patients

**DOI:** 10.1186/s43055-021-00519-9

**Published:** 2021-06-01

**Authors:** Ahmed M. Magdy, Mohammad A. Saad, Ahmed F. El Khateeb, Mona I. Ahmed, Dina H. Gamal El-Din

**Affiliations:** 1 Radiology Department, Faculty of Medicine, Fayoum University, Fayoum, Egypt; 2 Department of Critical Care, Faculty of Medicine, Fayoum University, Fayoum, Egypt; 3 Department of Chest Disease and Tuberculosis, Faculty of Medicine, Fayoum University, Fayoum, Egypt; 4 Radiology Department, Faculty of Medicine, Cairo University, Cairo, Egypt

**Correction to: Egypt J Radiol Nucl Med 52, 114 (2021)**

**https://doi.org/10.1186/s43055-021-00493-2**

Following publication of the original article [1], the authors identified errors in the affiliations. The correct assigned affiliations to the authors are given below.

Ahmed M. Magdy1*, Mohammad A. Saad1, Ahmed F. El Khateeb2, Mona I. Ahmed3 and Dina H. Gamal El-Din4

1Radiology department, Faculty of Medicine, Fayoum University.

2Department of critical care, Faculty of Medicine, Fayoum University.

3Department of chest disease and tuberculosis, Faculty of Medicine, Fayoum University.

4Radiology department, Faculty of Medicine, Cairo University.

The author group has been updated above and the original article [1] has been corrected.
